# Supplementary material for: Sequential actions of EOMES and T-BET promote stepwise maturation of natural killer cells
Source: Nat Commun. 2021 Sep 14;12:5446. doi: 10.1038/s41467-021-25758-2 (PMC8440589; doi:10.1038/s41467-021-25758-2)
Supplement: Supplementary file 1 — Supplementary Information [file 41467_2021_25758_MOESM1_ESM.pdf]

Supplementary information for

Sequential actions of Eomes and T-bet promote stepwise maturation of Natural Killer cells

Jiang Zhang[1,2], Stéphanie Le Gras[3,4], Kevin Pouxvielh[1], Fabrice Faure[5], Lucie Fallone[1], Nicolas Kern[1], Marion Moreews[1], Anne-Laure Mathieu[1], Raphaël Schneider[6], Quentin Marliac[1], Mathieu Jung[3,4], Aurore Berton[1], Simon Hayek[7], Pierre-Olivier Vidalain[1,7], Antoine Marçais[1], Garvin Dodard[8], Anne Dejean[9], Laurent Brossay[8], Yad Ghavi-Helm[6], and Thierry Walzer[1]

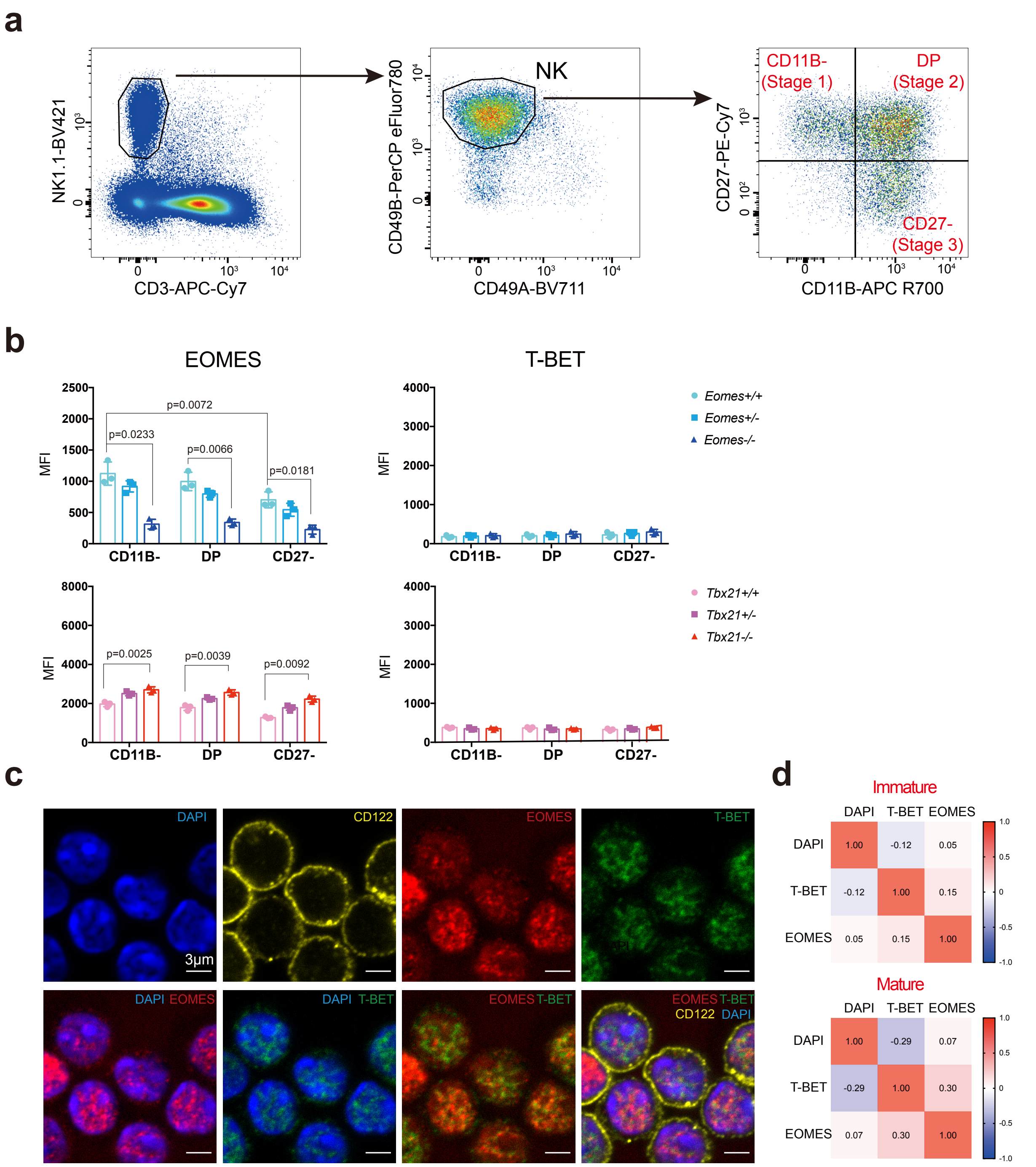

Supplementary Figure 1. (a) Gating strategy of NK cells in this study. NK cells were defined as NK1.1 +CD3-CD49B+CD49A-. NK cell subsets CD11B-, DP and CD27- were gated as shown in the right panel. **(b)** Flow cytometry measurement of T-BET and EOMES in gated NK cells from WT (*Tbx21*$^{+/+}$), *Tbx21*$^{+/-}$, *Tbx21*$^{-/-}$, *Ncr1*$^{Cre/+}$ *(Eomes*$^{+/+}$*)*, *Ncr1*$^{Cre/+}$ *X Eomes*$^{lox/+}$ (*Eomes*$^{+/-}$) and *Ncr1*$^{Cre/+}$ *X Eomes*$^{lox/lox}$ (*Eomes*$^{-/-}$) mice, as indicated. Bar graphs show the mean +/-SD fluorescence intensity (MFI) of EOMES and T-BETstaining in gated NK cell subsets from the BM. Data are representative of 3 experiments and 3 mice are shown for each group. **(c)** Mature NK cells (CD27-) were sorted from WT mice and subsequently stained for nucleus (DAPI), T-BET, EOMES and CD122 for confocal microscopy analysis. Shown are repre-sentative images of each staining and combinations for mature NK cells. Images are representative of three individual experiments. **(d)** Image-J analysis of fluorescence distribution showing the correlation between EOMES, T-BET and DAPI stainings in immature and mature NK cells. Unpaired t-tests (two-tailed) were used for statistical analysis of data presented in this figure.

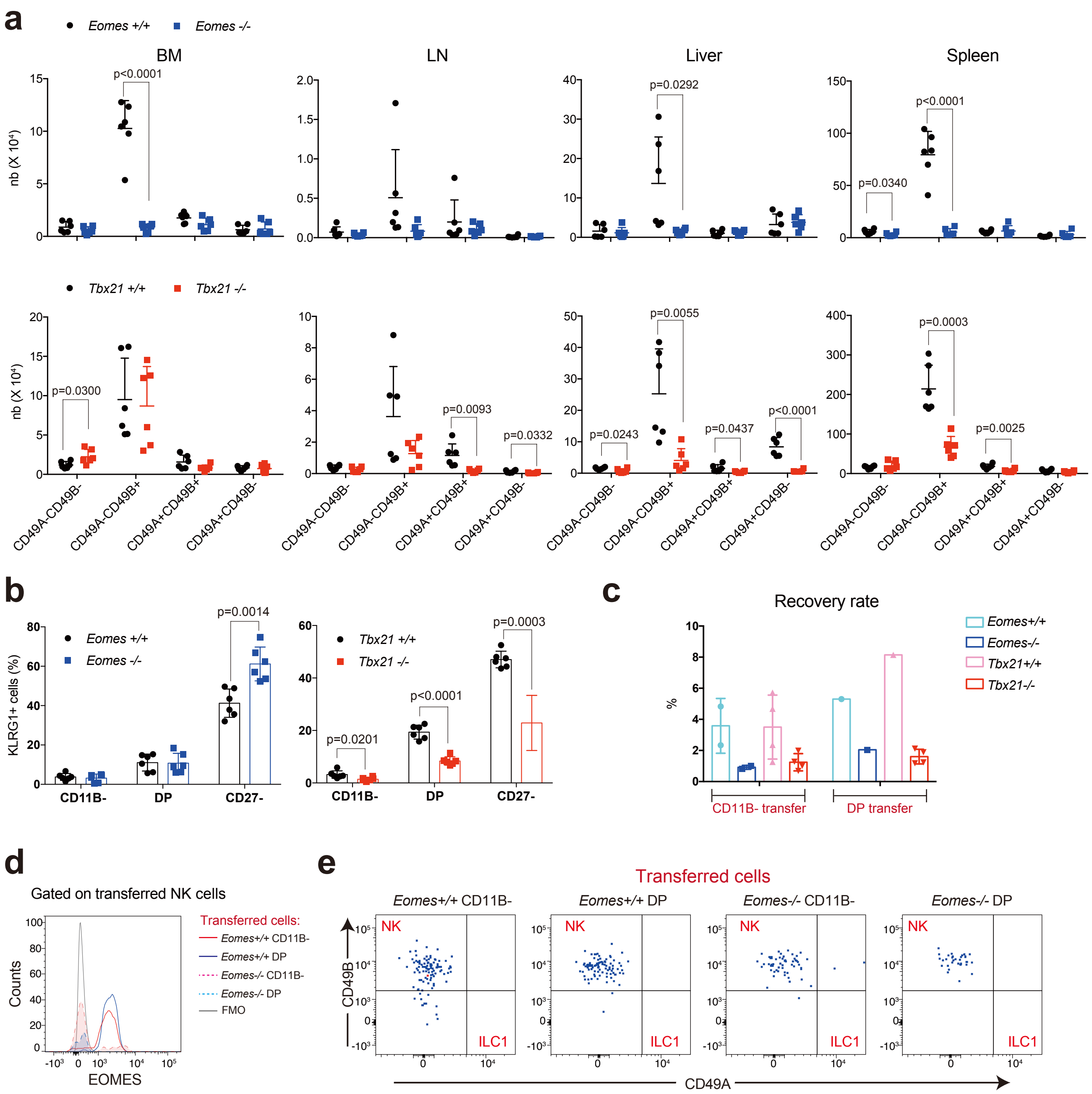

Supplementary Figure 2. (a) Number of NK1.1+CD3- ILCs of the CD49A+ CD49B-, CD49A+CD49B+, CD49A-CD49B+ and CD49A-CD49B- phenotype in the indicated organs of WT, *NK-Eomes*$^{-/-}$ and *Tbx21*$^{-/-}$ mice. **(b)** FACS measurement of KLRG1 expression in gated spleen NK cells from mice of the indicated genotype. Bar graphs show the mean +/-SD and dots corresponding to individual mice. Data are polled from 2 experiments with n=4-6 mice in total. **(c-e)** Relative to Figure 2d: **(c)** Recovery rate of transferred NK cells of the indicated genotype in the spleen of recipient mice. Bar graphs show the mean +/-SD. Data are from 1-4 mice repre-sentative of two experiments. **(d-e)** Flow cytometry analysis of EOMES **(d)** and CD49A/CD49B **(e)** expression in recovered NK cells of different genotypes, as indicated. Unpaired t-tests (two-tailed) were used for statistical analy-sis of data presented in this figure.

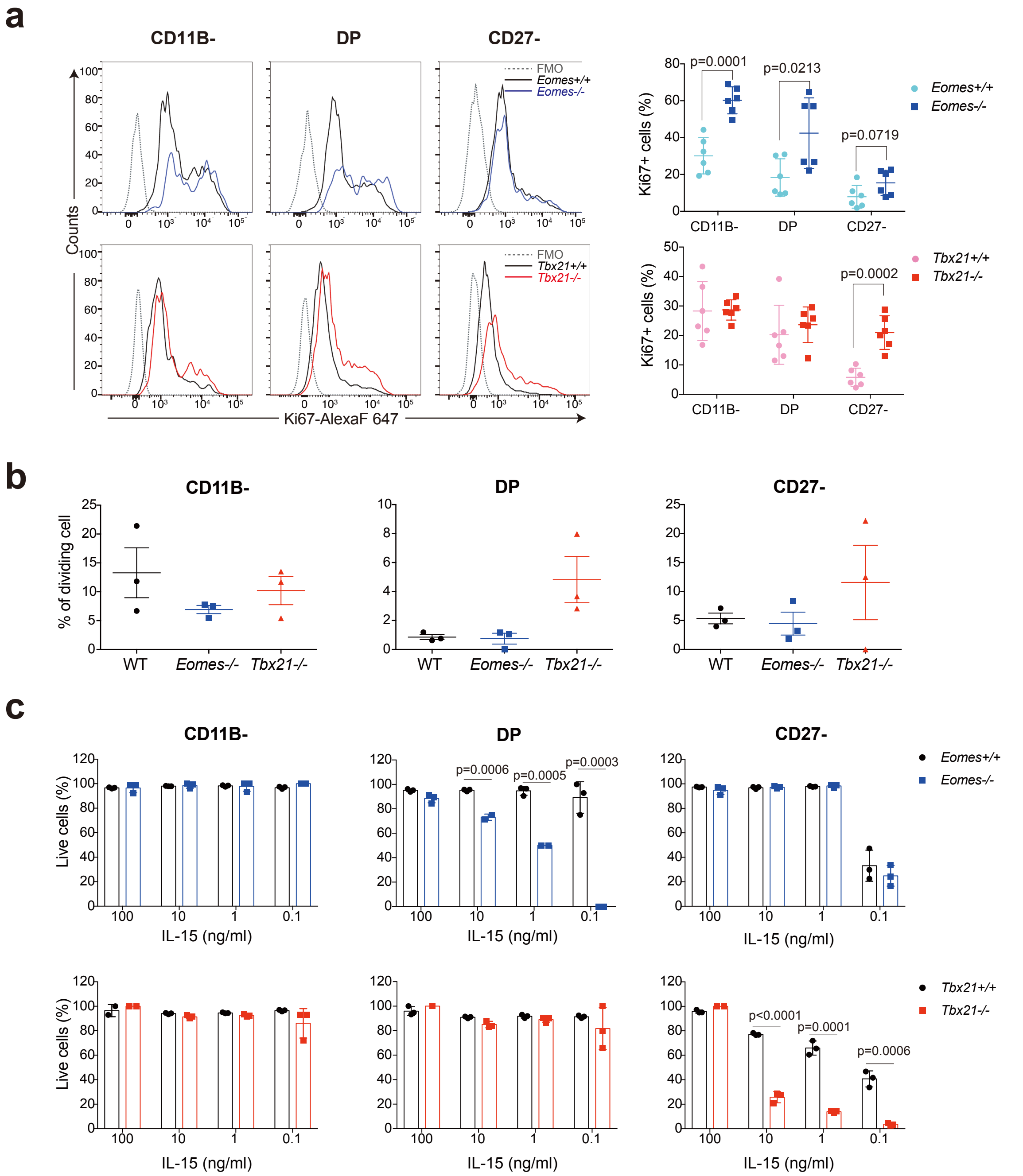

Supplementary Figure 3. Analysis of *Tbx21*$^{-/-}$ and *Eomes*$^{-/-}$ NK cell proliferation. (a) Flow cytometry analysis of Ki67 expression in spleen NK cells. Each dot corresponds to a single mouse (n=6, pooled from 2 experiments), and graphs show the mean percentage +/-SD. Representative FACS histograms are also shown. **(b)** Adoptive transfer experiment of CTV-labeled WT and *Tbx21*$^{-/-}$ or WT and *Eomes*$^{-/-}$ NK cells in Ly5a mice. Graphs show the mean percentage +/-SD of NK cells of each subset and of each genotype that underwent division two weeks after transfer, as determined by flow cytometry measurement of CTV dilution. (n=2 experiments, 3 mice each). **(c)** Analysis of subset viability after culturing with IL-15 for 48h at the indicated concentrations. Data are representative of 2 independent experiments. Unpaired t-tests (two-tailed) were used for statistical analysis of data presented in this figure.

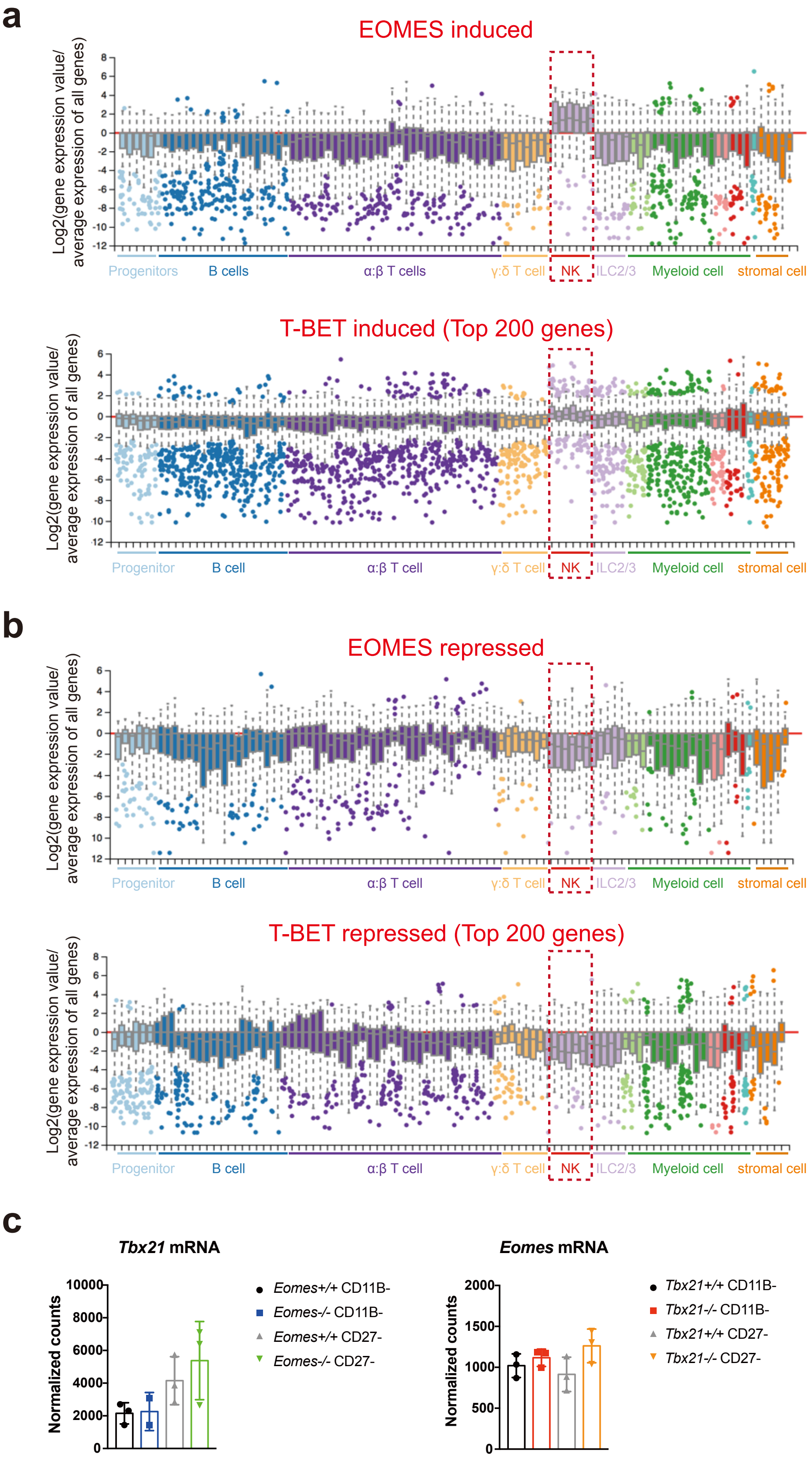

Supplementary Figure 4. Expression pattern of NK cell genes regulated by T-BET or EOMES across the immune system. **(a)** Expression profiles of NK cell genes activated by T-BET or EOMES across immune subsets (Immgen data[38]). **(b)** Expression profiles of NK cell genes repressed by T-BET or EOMES across immune subsets (Immgen data[38]). **(c)** Normalized read counts for T-BET or EOMES mRNA. Bar graphs show the mean +/-SD count extracted from RNA-seq experiment described in Figure 4. N=3 mice.

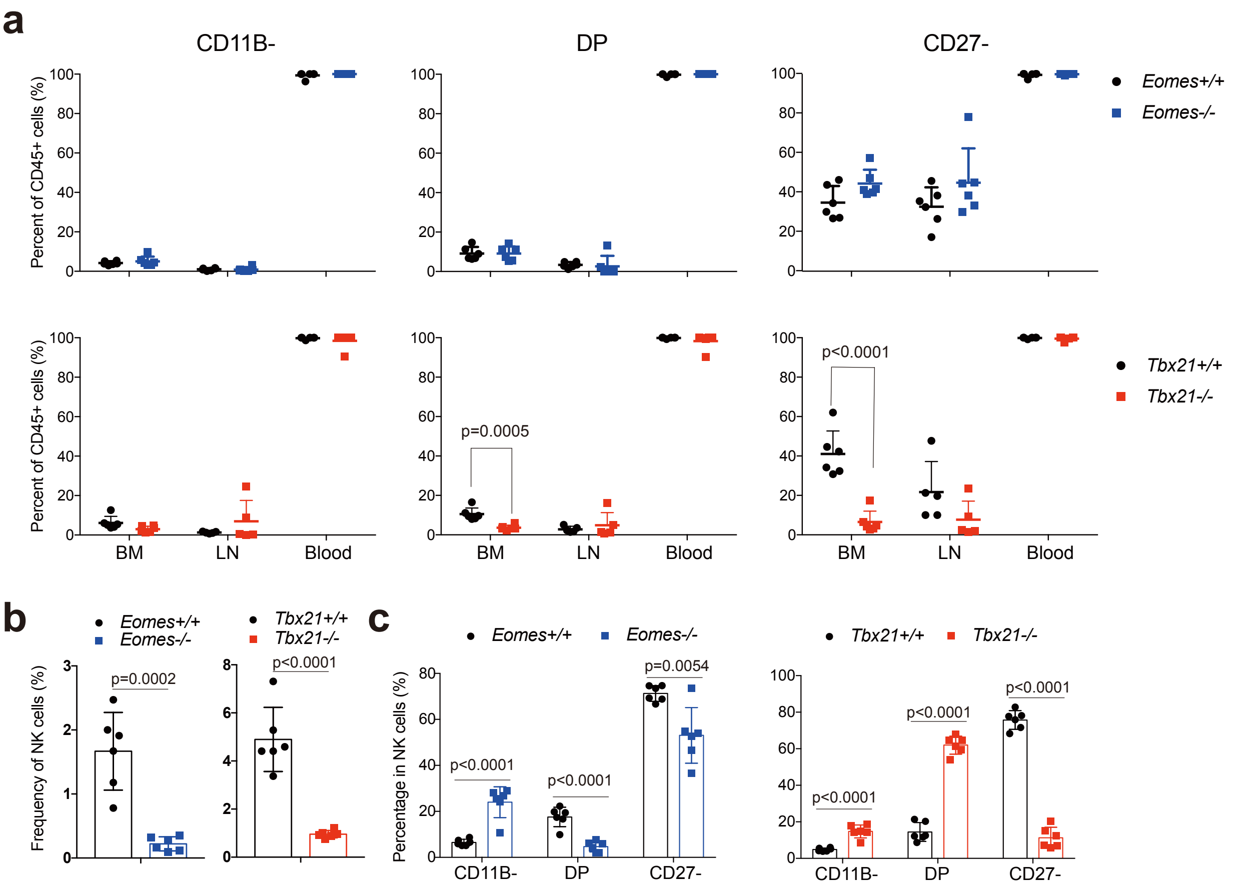

Supplementary Figure 5. T-BET promotes NK cell blood circulation. (a) Mice of the indicated genotypes were injected with CD45 MAb intravenously 5 minutes before sacrifice. Graphs show the mean +/-SD percentage of CD45+ NK cells among different subsets of different organs as determined by flow cytometry. Results were pooled from two experiments with n=5-6 mice in total. **(b-c)** Graphs show the mean +/-SD frequency of blood **(b)** NK cells and **(c)** NK cell subsets in the indicated genotype (n=6 pooled from two experiments). Unpaired t-tests (two-tailed) were used for statistical analysis of data presented in this figure.

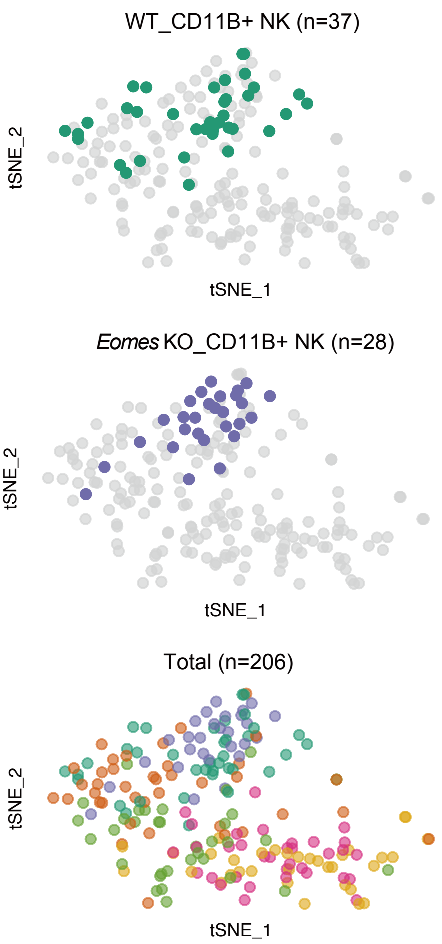

Supplementary Figure 6. **scRNAseq analysis of WT and *Eomes-/-* NK cells and ILC1s.** tSNE visualization of 300 single cell transcriptomes for the indicated sorted cell types. This figure shows the distribution of the samples based on the expression of the most variable genes in the dataset.

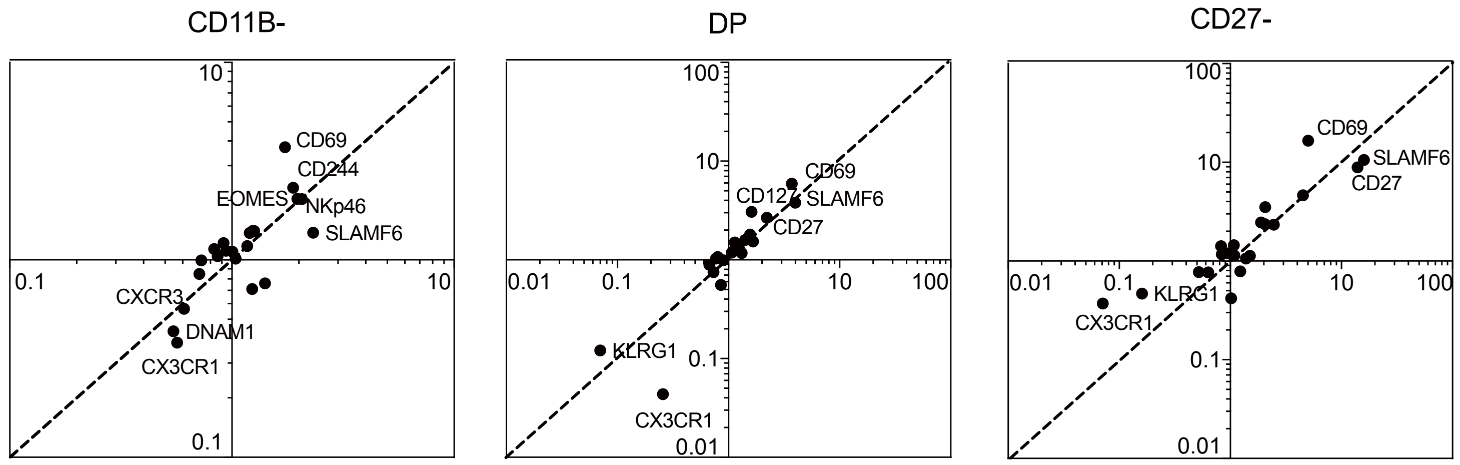

Supplementary Figure 7. The role of T-BET in NK cell differentiation is mostly cell-intrinsic. Sublethally irradiated mice Ly5a were reconstituted with a 1:1 mix of BM from Ly5a and *Tbx21*$^{-/-}$ mice to generate BM chimeric mice. 8 weeks later, spleen NK cells from these mice and from individual Ly5a and *Tbx21*$^{-/-}$ mice were analyzed by flow cytometry for a series of 23 parameters (DNAM1, NK1.1, CD27, CD11b, CD69, CXCR3, CD122, KLRG1, SLAMF6, CD127CD49b, CD160, NKp46, CX3CR1, Ki67, TCF7, EOMES, T-BET, CD244, CD11A, CRACC, GZMA, GZMB. Results show the mean expression ratio between KO and control NK cells for each marker and plotted as the ratio found in BM chimera mice as a function of that in individual mice. N=6 mice in each group. Unpaired t-tests (two-tailed) were used for statistical analysis of data presented in this figure.

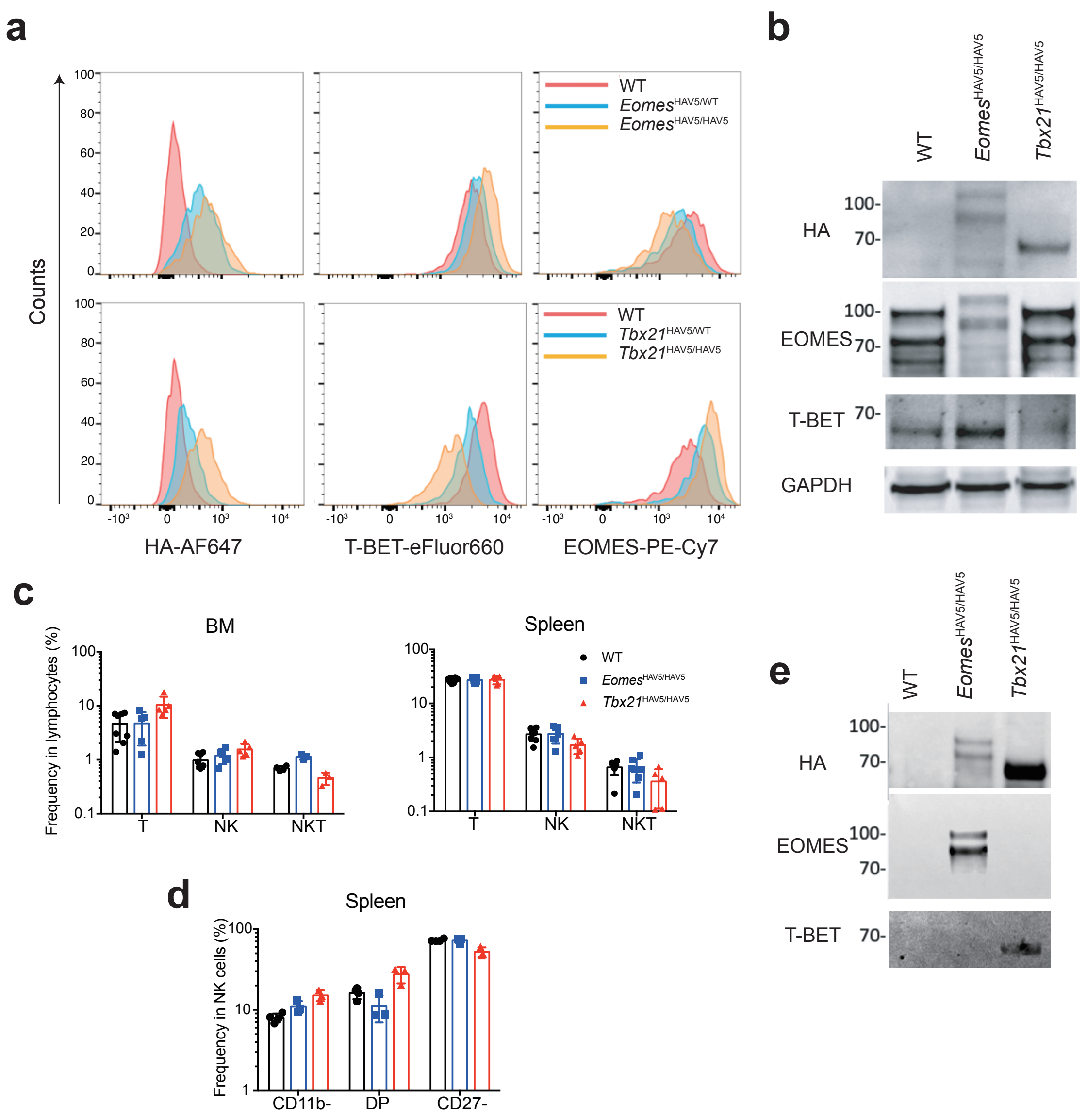

Supplementary Figure 8. Validation of HA-V5 expression in knock-in mice. (a) Flow cytometry analysis of T-BET, EOMES, and HA expression in gated NK cells from the indicated mouse strains. **(b)** WB analysis of HA, T-BET and EOMES expression in NK cells enriched from the spleen of the indicated mouse strains. **(c-d)** Flow cytometry analysis of NK cell frequency (mean+/-SD) in the indicated organs **(c)** and spleen NK cell maturation **(d)** in WT, *Tbx21-HAV5* and *Eomes-HAV5* mice. N=5-7 mice in each group pooled from two experiments. (e) Spleen cells from WT, *Eomes-HAV5* and *T-bet-HAV5* were lysed and the lysate was subjected to anti-HA IP. The immunoprecipitate was then western blotted with with anti-HA, anti-EOMES and anti-T-BET antibodies. Data are representative of two experiments.Unpaired t-tests (two-tailed) were used for statistical analysis of data presented in this figure.

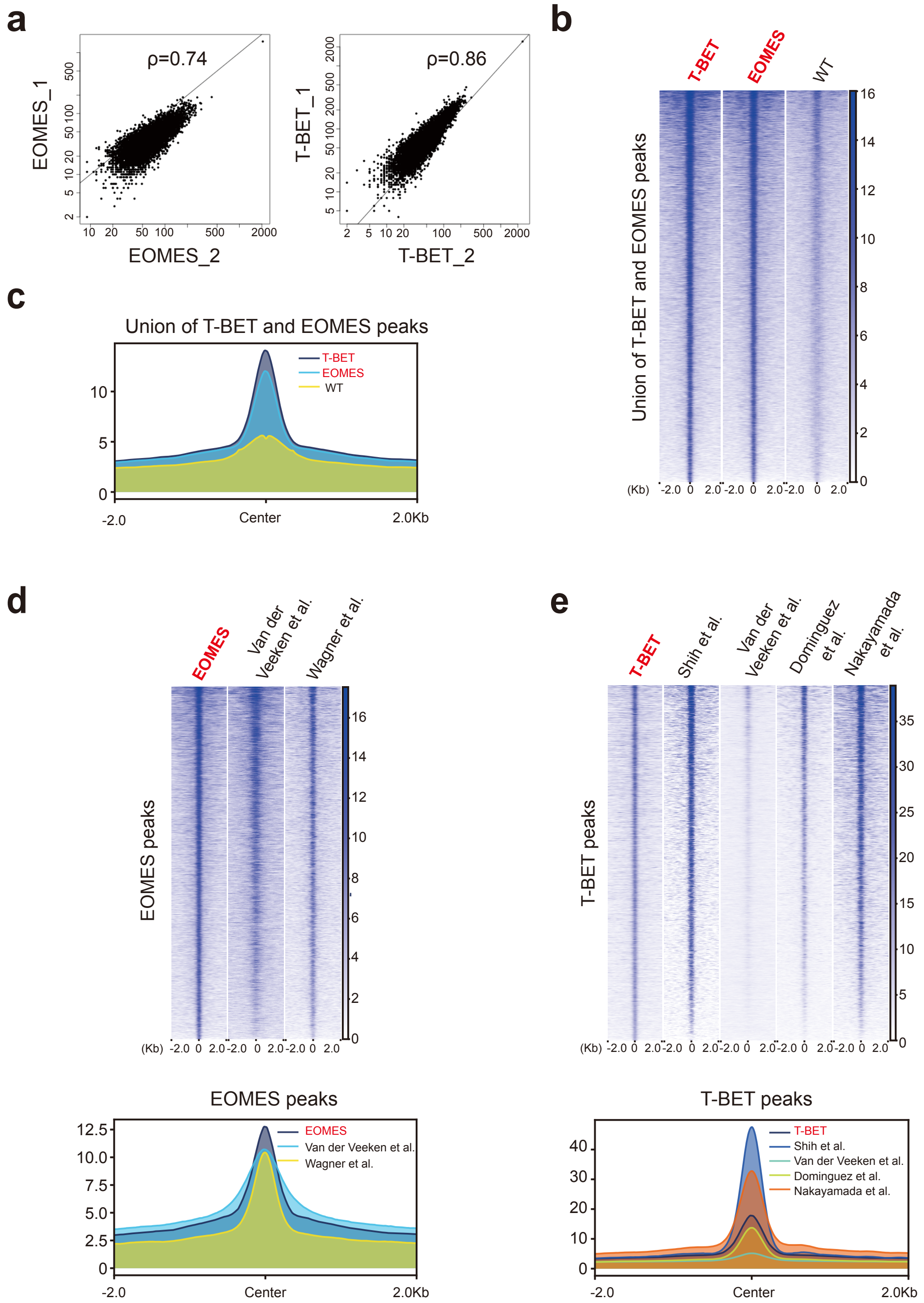

Supplementary Figure 9. Quality controls of EOMES and T-BET ChIP-seq experiments. (a) Scatter plot showing the number of reads in detected peaks in both replicates of EOMES and T-BET ChIP-seq experiments. The Spearman correlation coefficient is shown. **(b)** Heatmap showing EOMES and T-BET ChIP-seq data compared to controls in WT NK cells at the union of EOMES and T-BET peaks. Regions shown are centered at the middle of the peak and extended by +/- 2Kb. Data are the pool of both replicates for each condition. **(c)** Mean profile plots at the union of EOMES and T-BET ChIP-seq peaks +/-2Kb around the center of the regions. The control condition is also shown. **(d-e)** Heatmaps and mean profile plots showing read density at positions of EOMES **(d)** and T-BET **(e)** peaks as defined in our study, for different ChIP-seq experiments as follows: anti-EOMES: Van Der Veeken[59], Wagner[32]; anti-T-BET: Shih[15], Dominguez[61], Van Der Veeken[59], Nakayamada[62].

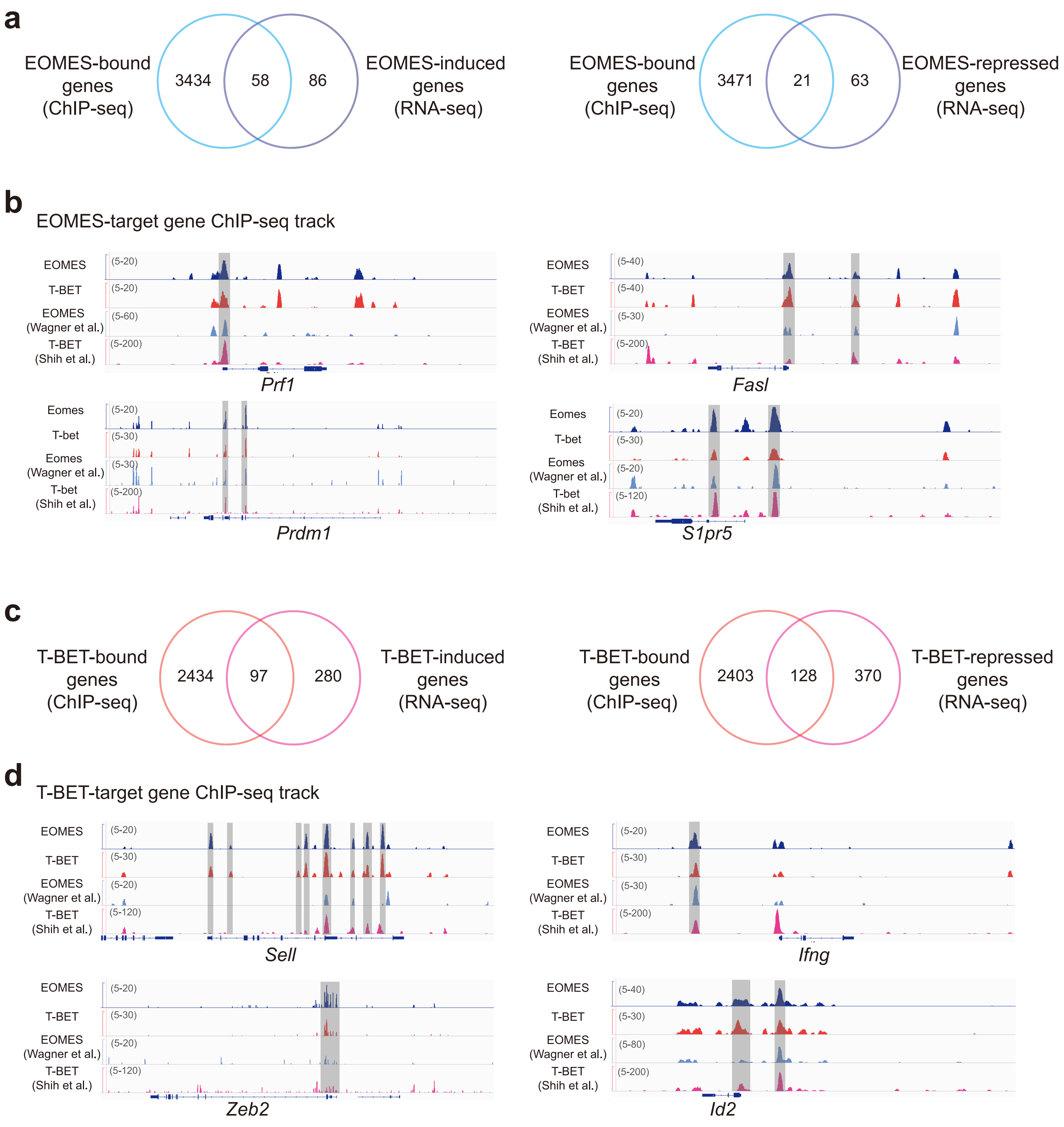

Supplementary Figure 10 related to Figure 8. EOMES and T-BET target genes. (a) Venn diagrams showing the overlap between EOMES-bound (ChIP-seq) and EOMES-induced or repressed genes (RNA-seq). **(b)** Examples of ChIP-seq tracks for selected EOMES target genes. **(c)** Venn diagrams showing the overlap between T-BET-bound (ChIP-seq) and T-BET-induced or repressed genes (RNA-seq). **(d)** Examples of ChIP-seq tracks for selected T-BET target genes. For **(a)** and **(c)** panels, p-values were calculated using the hypergeometric test and were p=2.930306e-38 and p=1.593572e-07 respectively.

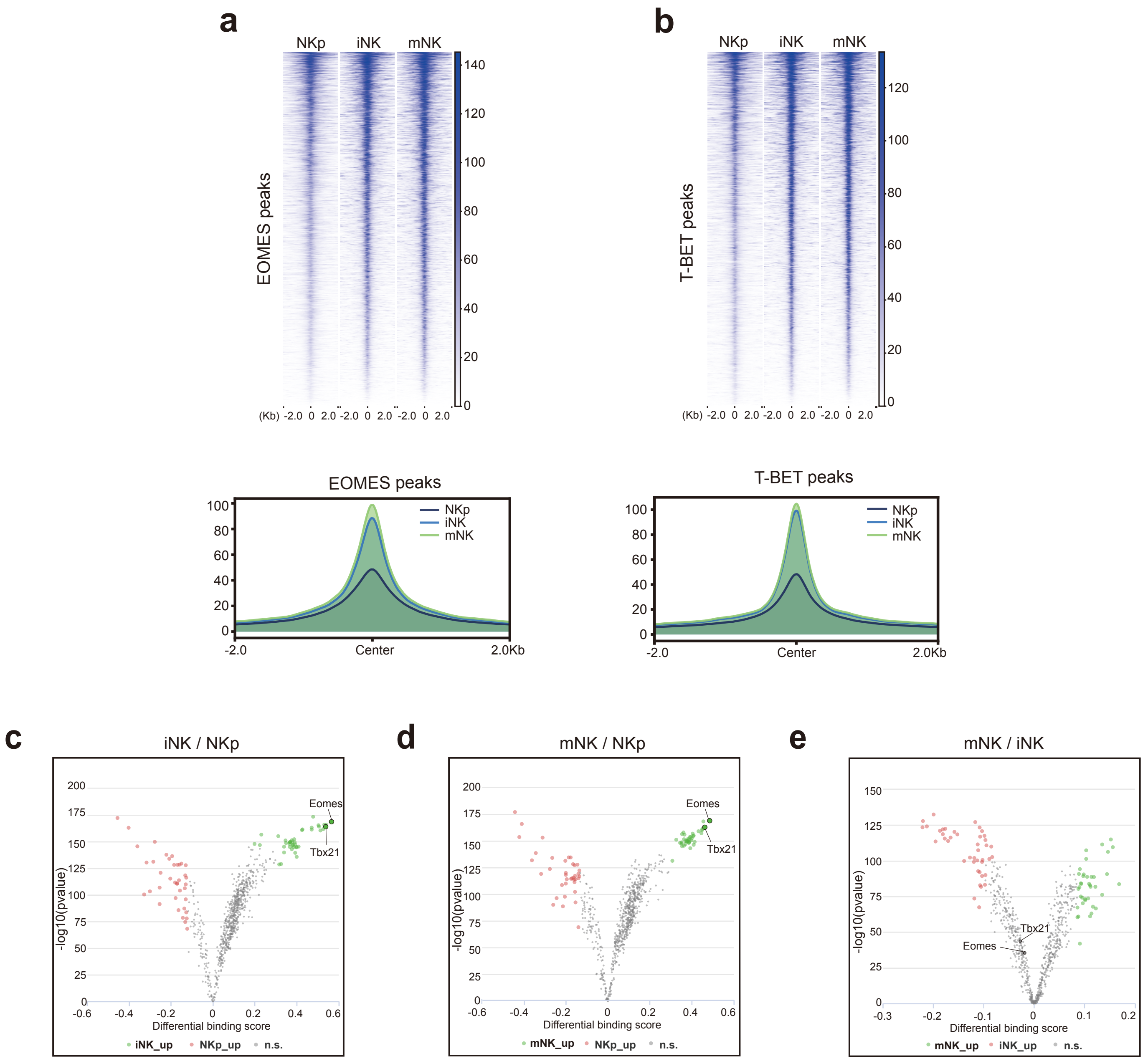

Supplementary Figure 11. T-BET/EOMES binding is associated with epigenetic changes during NK cell differentiation. (a-b) Heatmaps and mean profile plots of ATAC-seq regions for the indicated samples at EOMES **(q)** and T-BET **(b)** ChIP-seq peak positions. **(c-e)** Volcano plots showing differential TF binding sites as predicted by TOBIAS analysis of footprints in ATAC-seq data of NKp, iNK and mNK from Shin et al[15]. **(c)** NKp vs iNK; **(d)** NKp vs mNK; **(e)** iNK vs mNK.

Supplementary Table 1: Antibody list

**For Flow Cytometry Antibodies:**	Clone	Manufacturer	Catalogue	Dilution
Anti-mouse CD3e-FITC	145-2C11	Biolegend	Cat# 100306,	1/200
Anti-mouse CD3e-PE	145-2C11	eBiosciences,	Cat# 12-0031	1/400
Anti-mouse CD3-APC-Cy7	17A2	Biolegend	Cat# 100222	1/100
Anti-mouse CD3-AlexaF 700	17A2	Biolegend	Cat# 100216	1/100
Anti-mouse CD3-PerCP-eFluo710	17A2	eBiosciences	Cat# 46-0032-80	1/400
Anti-mouse CD11b-APC-R700	M1/70	Biolegend	Cat# 124216	1/800
Anti-mouse CD11b-APC-Cy7	M1/70	BD Biosciences	Cat# 557657	1/200
Anti-mouse CD11b-PE-Dazzle 594	M1/70	Biolegend	Cat# 101255	1/800
Anti-mouse CD19-APC-eF780	1D3	eBiosciences	Cat# 47-0193	1/200
Anti-mouse CD19-FITC	1D3	BD Biosciences	Cat# 553785	1/200
Anti-mouse CD27-PE	LG.7F9	eBiosciences	Cat# 12-0271-82	1/400
Anti-mouse CD27-PE-Cy7	LG.3A10	Biolegend	Cat# 124216	1/800
Anti-mouse CD27-BUV737	LG.3A10	BD Biosciences	Cat# 565307	1/100
Anti-mouse CD45-PE	30-F11	BD Biosciences	Cat# 553081	1/200
Anti-mouse CD45.1- BUV737	A20	BD Biosciences	Cat# 564574	1/200
Anti-mouse CD45.2-FITC	104	eBiosciences	Cat# 11-0454	1/400
Anti-mouse CD45.2-BV650	104	Biolegend	Cat# 109836	1/200
Anti-Mouse CD45.2-BV786	104	BD Biosciences	Cat# 563686	1/100
Anti-mouse CD49a-PE	Ha31/8	BD Biosciences	Cat# 562115	1/400
Anti-mouse CD49a- BV711	Ha31/8	BD Biosciences	Cat# 5648631	1/100
Anti-mouse CD49b-PerCP-eFluor710	DX5	eBiosciences	Cat# 46-5971	1/200
Anti-mouse CD49b-BV421	DX5	BD Biosciences	Cat# 563063	1/100
Anti-Mouse CD62L-PE Cy7	Mel14	eBiosciences	Cat# 25-0621-81	1/200
Anti-Mouse CD69-PE Cy7	H1.2F3	BD Biosciences	Cat# 561930	1/100
Anti-Mouse CD122-APC	TM-b1	Biolegend	Cat# 123214	1/200
Anti-Mouse CD127-APC eFluor780	A7R34	eBiosciences	Cat# 47-1271,	1/200
Anti-Mouse CD226-APC	10E5	eBiosciences	Cat# 17-2261-82	1/200
Anti-Mouse CD244.2 (2B4)-AlexaF 647	m2B4 (B6)45	Biolegend	Cat# 133510	1/200
Anti-Mouse Eomes-PE-Cy7	Dan11mag	eBiosciences	Cat# 25-4875-80	1/100
Anti-Mouse GzmA-PE-Cy7	3G8.5	eBiosciences	Cat# 25-5831-82	1/200
Anti-Mouse IFNγ-PE-Cy7	Dan11mag	eBiosciences	Cat# 25-4875-80	1/200
Anti-Mouse Ki67-AlexaF 647	SolA15	eBiosciences	Cat# 51-5698	1/100
Anti-Mouse KLRG1-BUV395	2F1	BD Biosciences	Cat# 740279	1/100
Anti-Mouse Ly49A-FITC	YE1/48.10.6	Biolegend	Cat# 116805,	1/200
Anti-Mouse Ly49D-VioGreen	4 E5	Miltenyi	Cat# 130-102-206	1/100
Anti-Mouse Ly49G2-BUV395	4D11	BD Biosciences	Cat# 742885	1/200
Anti-mouse NK1.1-APC	PK136	BD Biosciences	Cat# 550627	1/400
Anti-mouse NK1.1-BV421	PK136	Biolegend	Cat# 108732	1/400
Anti-mouse NKp46-AlexaF 647	29A1.4	BD Biosciences	Cat# 560755	1/100
Anti-mouse NKp46-V450	29A1.4	BD Biosciences	Cat# 560764	1/100
Anti-Mouse phospho-STAT4	38/p-Stat4	BD Biosciences	Cat# 558249	1/40
Anti-Mouse T-bet-FITC	4B10	Biolegend	Cat# 644812	1/50
Anti-Mouse T-bet-PE-Cy7	4B10	eBiosciences	Cat# 25-5825-80	1/200
Anti-Mouse T-bet-eFluor 660	4B10	eBiosciences	Cat# 50-5825	1/100
Anti-HA-Tag-AF647	6E2	Cell Signaling T	Cat# 3444	1/200

**For Western Blot Antibodies:**				
Anti-Mouse GAPDH	14C10	Cell Signaling T	**Cat# 2118L**	1/1000
Anti-Mouse T-bet	4B10	Biolegend	Cat# 644801	1/200
Anti-Mouse Eomes	polyclonal	Abcam	Cat# ab23345	1/100
Anti-HA-Tag	C29F4	Cell Signaling T	Cat #3724	1/1000

Supplementary Table 2: Primer list

Primer name	sequence
Ex6-F1	GCGAAGGAGACACTAAGAGGAGGAG
Ex6-R1	(AGCACCAGGTTCGTGACTGTAGTTC
Ex6-F2	GCGAAGGAGACACTAAGAGGAGGAG
Ex6-R2	AGCACCAGGTTCGTGACTGTAGTTC

Supplementary Methods

Analysis of public data

ChIP-seq datasets. The following published datasets were downloaded from GEO or SRA in SRA format and converted to FASTQ format using the fastq-dump program in the sratoolkit (version 2.1.9).

**Sample name**	**GEO/SRA accession**	**Publication**
87_T-bet_Sp_NK	GSM2056378	Shih et al.
Tbet_WT_Th1	GSM836124	Nakayamada et al.
CD8_TBET_WT.1	SRX1070596	Dominguez et al
Eomes_ChIPseq_rep1	GSM3900380	Wagner et al.
b6_cast_lcmv_cd8_d07_chip_r1_eomes	GSM3612595	van der Veeken et al.
b6_cast_lcmv_cd8_d07_chip_r2_tbet	GSM3612599	van der Veeken et al.

Reads were mapped to the Mus musculus genome (assembly mm10) using Bowtie v1.0.0 with default parameters except for “-m 1 --strata --best”.

ATAC-seq datasets. The following published datasets were downloaded from GEO or SRA in SRA format and converted to FASTQ format using the fastq-dump program in the sratoolkit (version 2.1.9).

**GEO Identifier**	**Sample name**	**Condition**
SRR3152814	9_ATAC_BM_iNK_rpt1	iNK
SRR3152815	10_ATAC_BM_iNK_rpt2	iNK
SRR3152822	17_ATAC_BM_NKp_rpt1	NKp
SRR3152823	18_ATAC_BM_NKp_rpt2	NKp
SRR3152848	43_ATAC_Sp_NK_rpt1	Sp_NK
SRR3152849	44_ATAC_Sp_NK_rpt2	Sp_NK

These data were analyzed using the Encode ATAC-seq pipeline v1.5.1. Briefly, bowtie v2.3.4.3 was used to align reads to the reference mouse genome mm10/GRCh38 and MACS2 v2.2.4 was used to call OCRs. Footprinting analysis of ATAC-seq data was performed using the suite of tools TOBIAS [51]. Briefly, TOBIAS ATACorrect corrects Tn5 insertion bias, TOBIAS ScoreBigwig calculates footprint scores within regulatory regions, TOBIAS BINDetect estimates bound/unbound transcription factor binding sites. Known motifs were from the JASPAR CORE (2020) for vertebrate non-redundant database.
