# Supplementary material for: Sequential actions of EOMES and T-BET promote stepwise maturation of natural killer cells
Source: Nat Commun. 2021 Sep 14;12:5446. doi: 10.1038/s41467-021-25758-2 (PMC8440589; doi:10.1038/s41467-021-25758-2)
Supplement: Supplementary file 3 — Description of Additional Supplementary Files [file 41467_2021_25758_MOESM3_ESM.pdf]

Description of Additional Supplementary Files

Supplementary Data 1. Genes differentially expressed in *Tbx21*$^{-/-}$, or *NK-Eomes*$^{-/-}$ immature or mature NK cells compared to controls. The list is ranked from top to bottom based on the number of comparisons for which differential expression was measured. Log2 fold changes and adjusted p-value for the comparisons are shown.

Supplementary Data 2. List of validated T-BET and EOMES target genes, *i.e.* genes for which DNA binding was measured for T-BET or EOMES, and for which differential expression was measured (either up or down) in *Tbx21*$^{-/-}$, or *NK-Eomes*$^{-/-}$ immature or mature NK cells compared to controls, respectively.
