# Supplementary material for: Sequential actions of EOMES and T-BET promote stepwise maturation of natural killer cells
Source: Nat Commun. 2021 Sep 14;12:5446. doi: 10.1038/s41467-021-25758-2 (PMC8440589; doi:10.1038/s41467-021-25758-2)
Supplement: Supplementary file 7 — Reporting Summary [file 41467_2021_25758_MOESM7_ESM.pdf]

nature research

Corresponding author(s): Thierry Walzer
Last updated by author(s): May 17, 2021

Reporting Summary

Nature Research wishes to improve the reproducibility of the work that we publish. This form provides structure for consistency and transparency in reporting. For further information on Nature Research policies, see our Editorial Policies and the Editorial Policy Checklist.

Statistics

For all statistical analyses, confirm that the following items are present in the figure legend, table legend, main text, or Methods section.

n/a	Confirmed	
☐	☒	The exact sample size (n) for each experimental group/condition, given as a discrete number and unit of measurement
☐	☒	A statement on whether measurements were taken from distinct samples or whether the same sample was measured repeatedly
☐	☒	The statistical test(s) used AND whether they are one- or two-sided<br>*Only common tests should be described solely by name; describe more complex techniques in the Methods section.*
☒	☐	A description of all covariates tested
☐	☒	A description of any assumptions or corrections, such as tests of normality and adjustment for multiple comparisons
☒	☐	A full description of the statistical parameters including central tendency (e.g. means) or other basic estimates (e.g. regression coefficient) AND variation (e.g. standard deviation) or associated estimates of uncertainty (e.g. confidence intervals)
☐	☒	For null hypothesis testing, the test statistic (e.g. F, t, r) with confidence intervals, effect sizes, degrees of freedom and P value noted<br>*Give P values as exact values whenever suitable.*
☒	☐	For Bayesian analysis, information on the choice of priors and Markov chain Monte Carlo settings
☒	☐	For hierarchical and complex designs, identification of the appropriate level for tests and full reporting of outcomes
☒	☐	Estimates of effect sizes (e.g. Cohen's d, Pearson's r), indicating how they were calculated

Our web collection on statistics for biologists contains articles on many of the points above.

Software and code

Policy information about availability of computer code

Data collection

Flow Cytometry data was collected with BD FACSDIVA

ChIP-seq datasets. The following published datasets were downloaded from GEO or SRA in SRA format and converted to FASTQ format using the fastq-dump program in the sratoolkit (version 2.1.9).
Sample name GEO/SRA accession Publication
87_T-bet_Sp_NK GSM2056378 Shih et al.
Tbet_WT_Th1 GSM836124 Nakayamada et al.
CD8_TBET_WT.1 SRX1070596 Dominguez et al
Eomes_ChIPseq_rep1 GSM3900380 Wagner et al.
b6_cast_lcmv_cd8_d07_chip_r1_eomes GSM3612595 van der Veeken et al.
b6_cast_lcmv_cd8_d07_chip_r2_tbet GSM3612599 van der Veeken et al.
Reads were mapped to the Mus musculus genome (assembly mm10) using Bowtie v1.0.0 with default parameters except for "-m 1 --strata --best".
ATAC-seq datasets. The following published datasets were downloaded from GEO or SRA in SRA format and converted to FASTQ format using the fastq-dump program in the sratoolkit (version 2.1.9).
GEO Identifier Sample name Condition
SRR3152814 9_ATAC_BM_iNK_rpt1 iNK
SRR3152815 10_ATAC_BM_iNK_rpt2 iNK
SRR3152822 17_ATAC_BM_NKp_rpt1 NKp
SRR3152823 18_ATAC_BM_NKp_rpt2 NKp
SRR3152848 43_ATAC_Sp_NK_rpt1 Sp_NK

SRR3152849 44_ATAC_Sp_NK_rpt2 Sp_NK

These data were analyzed using the Encode ATAC-seq pipeline v1.5.1. Briefly, bowtie v2.3.4.3 was used to align reads to the reference mouse genome mm10/GRCh38 and MACS2 v2.2.4 was used to call OCRs. Footprinting analysis of ATAC-seq data was performed using the suite of tools TOBIAS v0.12.4 51. Briefly, TOBIAS ATACorrect corrects Tn5 insertion bias, TOBIAS ScoreBigwig calculates footprint scores within regulatory
regions, TOBIAS BINDetect estimates bound/unbound transcription factor binding sites. Known motifs were from the JASPAR CORE (2020) for vertebrate non-redundant database.

Data analysis

Flow Cytometry data was analyzed with FlowJo Software (10.6.1, FlowJo LLC, BD Life Sciences) and Prism 7 (Graphpad Software)

Bio-informatic analysis (RNA-seq, ChIP-seq)
RNA-seq. Reads were processed using an in-house RNA-seq pipeline of GenomEast facility. Briefly: raw data were preprocessed using cutadapt 1.10 67 in order to remove adaptor and low-quality sequences (Phred quality score below 20). Reads shorter than 40 bp were removed for further analysis. Remaining reads were mapped to mouse rRNA sequences using bowtie 2.2.8 68 and reads mapped to rRNA sequences were discarded for further analysis. Remaining reads were aligned to mm10 assembly of the mouse genome with STAR 2.5.3a 69. Gene quantification was performed with htseq-count 0.6.1p1 70 using "union" mode and Ensembl 96 annotations. Differential gene expression analysis between groups of samples was performed using method implemented in the Bioconductor R package DESeq2 1.16.1 71, with the following non-default options: betaPrior=TRUE, alpha=0.05. P-values were adjusted for multiple testing using the Benjamini and Hochberg method.

ChIP-seq. The Encode Pipeline v1.6.1 was used to map reads and detect Tbet and Eomes peaks. Briefly, bowtie v2.3.4.3 68 was used to align reads to the reference mouse genome mm10/GRCh38. spp v1.15.5 72 was used to detect around 300,000 peaks and the IDR method was used to select reproducible peaks with an IDR threshold <= 0.05. The « IDR optimal » set of peaks was selected for downstream analysis. Controls are flag-HA chipped samples in WT NK cells. Peaks were annotated relative to genomic features using Homer annotatePeaks.pl v4.11.1 73. Annotations were extracted from Ensembl v96.

Reproducibility of ChIPs. We compared the number of reads in detected peaks in the replicates. BEDtools makewindows v2.26.0 74 was used to compute all non-overlapping 10Kb long bins along the mouse genome. BEDtools intersect was used to count the number of reads falling into each bin for all IP samples. Read counts per bin are presented in a scatterplot and the Spearman correlation coefficient is computed. Heatmaps were generated using Deeptools v3.5 75d using the tool bamCoverage to generate bigwigs files with a step of 10 nt. Bigwig files were normalized using the RPGC method. Then, the tool Deeptools computeMatrix v3.5 was used to generate a count matrix at the positions of interest and finally the tools Deeptools plotHeatmap v3.5 and plotProfile v3.5 were used to generate heatmaps and mean profile plots. Data presented on the heatmap and mean profile are pooled by condition.

Motif analysis. The tool FIMO 47 of the MEME suite v4.10 was used to detect the Tbox motif EOMES/MA0800.1 (source Jaspar). The tool MEME-chip v4.10 48 was used with default parameters except for « -meme-mod zoops -meme-nmotifs 20 -meme-minw 8 -meme-maxw 25 » to de
novo detect motifs in Tbet and Eomes peak sequences. We detected motifs in sequences located +/-100nt around peak summits.

For manuscripts utilizing custom algorithms or software that are central to the research but not yet described in published literature, software must be made available to editors and reviewers. We strongly encourage code deposition in a community repository (e.g. GitHub). See the Nature Research guidelines for submitting code & software for further information.

Data

Policy information about availability of data

All manuscripts must include a data availability statement. This statement should provide the following information, where applicable:

- Accession codes, unique identifiers, or web links for publicly available datasets
- A list of figures that have associated raw data
- A description of any restrictions on data availability

ChIP-seq and RNA-seq data that support the findings of this study have been deposited in the Geo repository with the accession code GSE168242 (https://www.ncbi.nlm.nih.gov/geo/query/acc.cgi?acc=GSE168242).

ChIP-seq datasets. The following published datasets were downloaded from GEO or SRA

Sample name GEO/SRA accession Publication
87_T-bet_Sp_NK GSM2056378 Shih et al.
Tbet_WT_Th1 GSM836124 Nakayamada et al.
CD8_TBET_WT.1 SRX1070596 Dominguez et al
Eomes_ChIPseq_rep1 GSM3900380 Wagner et al.
b6_cast_lcmv_cd8_d07_chip_r1_eomes GSM3612595 van der Veeken et al.
b6_cast_lcmv_cd8_d07_chip_r2_tbet GSM3612599 van der Veeken et al.

ATAC-seq datasets. The following published datasets were downloaded from GEO
GEO Identifier Sample name Condition
SRR3152814 9_ATAC_BM_iNK_rpt1 iNK
SRR3152815 10_ATAC_BM_iNK_rpt2 iNK
SRR3152822 17_ATAC_BM_NKp_rpt1 NKp
SRR3152823 18_ATAC_BM_NKp_rpt2 NKp

SRR3152848 43_ATAC_Sp_NK_rpt1 Sp_NK
SRR3152849 44_ATAC_Sp_NK_rpt2 Sp_NK

Field-specific reporting

Please select the one below that is the best fit for your research. If you are not sure, read the appropriate sections before making your selection.

- [x] Life sciences
- [ ] Behavioural & social sciences
- [ ] Ecological, evolutionary & environmental sciences

For a reference copy of the document with all sections, see nature.com/documents/nr-reporting-summary-flat.pdf

Life sciences study design

All studies must disclose on these points even when the disclosure is negative.

Sample size	We did not perform sample size calculations. Sample size was determined to be adequate based on the magnitude and consistency of measurable differences between groups, as well as feasibility of performing highly technical experiments with a rare cell population.<br>In all experiments NK cells were analyzed by flow cytometry, we performed at least three biological replicates for each group in each experiment. In general, the number of biological replicates largely exceeded this number. For RNAseq experiments, three replicates were used in each group. For the ChIP-seq experiment, we only used two replicates because this type of experiment is technically very challenging and requires lots of mice for a single experiment.
Data exclusions	We did not exclude data
Replication	All experiments were reproduced at least three times as stated above.
Randomization	This is not relevant in our experiment, as all comparisons were performed between defined groups ie control vs knockout mice.
Blinding	Blinding wass not performed during data collection. However, RNA-seq data and ATAC-seq samples were processed by separate scientists and each data set was analyzed by separate bioinformaticians.

Reporting for specific materials, systems and methods

We require information from authors about some types of materials, experimental systems and methods used in many studies. Here, indicate whether each material, system or method listed is relevant to your study. If you are not sure if a list item applies to your research, read the appropriate section before selecting a response.

Materials & experimental systems

n/a	Involved in the study	
☐	☒	Antibodies
☒	☐	Eukaryotic cell lines
☒	☐	Palaeontology and archaeology
☐	☒	Animals and other organisms
☒	☐	Human research participants
☒	☐	Clinical data
☒	☐	Dual use research of concern

Methods

n/a	Involved in the study	
☐	☒	ChIP-seq
☐	☒	Flow cytometry
☒	☐	MRI-based neuroimaging

Antibodies

Antibodies used

For Flow Cytometry Antibodies:
Anti-mouse CD3e-FITC (145-2C11), Biolegend, Cat# 100306, 1/200
Anti-mouse CD3e-PE (145-2C11), eBiosciences, Cat# 12-0031, 1/400
Anti-mouse CD3-APC-Cy7 (17A2), Biolegend, Cat# 100222, 1/100
Anti-mouse CD3-AlexaF 700 (17A2), Biolegend, Cat# 100216, 1/100
Anti-mouse CD3-PerCP-eFluo710 (17A2), eBiosciences, Cat# 46-0032-80, 1/400
Anti-mouse CD11b-APC-R700 (M1/70), Biolegend, Cat# 124216, 1/800
Anti-mouse CD11b-APC-Cy7 (M1/70), BD Biosciences, Cat# 557657, 1/200
Anti-mouse CD11b-PE-Dazzle 594 (M1/70), Biolegend, Cat# 101255, 1/800
Anti-mouse CD19-APC-eF780 (1D3), eBiosciences, Cat# 47-0193, 1/200
Anti-mouse CD19-FITC (1D3), BD Biosciences, Cat# 553785, 1/200
Anti-mouse CD27-PE (LG.7F9), eBiosciences, Cat# 12-0271-82, 1/400
Anti-mouse CD27-PE-Cy7 (LG.3A10), Biolegend, Cat# 124216, 1/800
Anti-mouse CD27-BUV737 (LG.3A10), BD Biosciences, Cat# 565307, 1/100
Anti-mouse CD45-PE (30-F11), BD Biosciences, Cat# 553081, 1/200

Anti-mouse CD45.1- BUV737 (A20), BD Biosciences, Cat# 564574, 1/200
Anti-mouse CD45.2-FITC (104), eBiosciences, Cat# 11-0454, 1/400
Anti-mouse CD45.2-BV650 (104), Biolegend, Cat# 109836, 1/200
Anti-Mouse CD45.2-BV786 (104), BD Biosciences, Cat# 563686, 1/100
Anti-mouse CD49a-PE (Ha31/8), BD Biosciences, Cat# 562115, 1/400
Anti-mouse CD49a- BV711 (Ha31/8), BD Biosciences, Cat# 5648631, 1/100
Anti-mouse CD49b-PerCP-eFluor710 (DX5), eBiosciences, Cat# 46-5971, 1/200
Anti-mouse CD49b-BV421 (DX5), BD Biosciences, Cat# 563063, 1/100
Anti-Mouse CD62L-PE Cy7 (Mel14), eBiosciences, Cat# 25-0621-81, 1/200
Anti-Mouse CD69-PE Cy7 (H1.2F3), BD Biosciences, Cat# 561930, 1/100
Anti-Mouse CD122-APC (TM-b1), Biolegend, Cat# 123214, 1/200
Anti-Mouse CD127-APC eFluor780 (A7R34), eBiosciences, Cat# 47-1271, 1/200
Anti-Mouse CD226-APC (10E5), eBiosciences, Cat# 17-2261-82, 1/200
Anti-Mouse CD244.2 (2B4)-AlexaF 647 (m2B4 (B6)458.1), Biolegend, Cat# 133510, 1/200
Anti-Mouse Eomes-PE-Cy7 (Dan11mag), eBiosciences, Cat# 25-4875-80, 1/100
Anti-Mouse GzmA-PE-Cy7 (3G8.5), eBiosciences, Cat# 25-5831-82, 1/200
Anti-Mouse IFNγ-PE-Cy7 (Dan11mag), eBiosciences, Cat# 25-4875-80, 1/200
Anti-Mouse Ki67-AlexaF 647 (SolA15), eBiosciences, Cat# 51-5698, 1/100
Anti-Mouse KLRG1-BUV395 (2F1), BD Biosciences, Cat# 740279, 1/100
Anti-Mouse Ly49A-FITC (YE1/48.10.6), Biolegend, Cat# 116805, 1/200
Anti-Mouse Ly49D-VioGreen (4 E5), Miltenyi, Cat# 130-102-206, 1/100
Anti-Mouse Ly49G2-BUV395 (4D11), BD Biosciences, Cat# 742885, 1/200
Anti-mouse NK1.1-APC (PK136), BD Biosciences, Cat# 550627, 1/400
Anti-mouse NK1.1-BV421 (PK136), Biolegend, Cat# 108732, 1/400
Anti-mouse NKp46-AlexaF 647 (29A1.4), BD Biosciences, Cat# 560755, 1/100
Anti-mouse NKp46-V450 (29A1.4), BD Biosciences, Cat# 560764, 1/100
Anti-Mouse phospho-STAT4 (Y693) -PE (38/p-Stat4), BD Biosciences, Cat# 558249, 1/40
Anti-Mouse T-bet-FITC (4B10), Biolegend, Cat# 644812, , 1/50
Anti-Mouse T-bet-PE-Cy7 (4B10), eBiosciences, Cat# 25-5825-80, 1/200
Anti-Mouse T-bet-eFluor 660 (4B10), eBiosciences, Cat# 50-5825, 1/100
Anti-HA-Tag-AF647 (6E2), Cell Signaling T, Cat# 3444, 1/200

For Western Blot Antibodies:
Anti-Mouse GAPDH (14C10), Cell Signaling T, Cat# 2118, 1/1000
Anti-Mouse T-bet (4B10), Biolegend, Cat# 644802, 1/200
Anti-Mouse Eomes, Abcam, Cat# ab23345, 1/100
Anti-HA-Tag (C29F4), Cell Signaling T, Cat# 3724, 1/1000

Validation

Antibodies were from commercial vendors as specified above. Specificity was based on manufacturers' provided description and data sheets, and have been validated by the manufacturers, as stated on their associated product pages. Antibodies were titrated on relevant cells and tissues prior to usage.

Animals and other organisms

Policy information about studies involving animals; ARRIVE guidelines recommended for reporting animal research

Laboratory animals

Wild-type C57BL/6, Ly5a mice were purchased from Charles River Laboratories (L'Arbresle). NCR1-iCre, Eomeslox/lox and Tbx21-/- mice were used. Some mice were intercrossed as described. T-bet HAV5 and Eomes HAV5 knock-in mice were generated in this study as well. Mice used in this study were 6-12 weeks of age. For BM chimera, mice were 8 weeks at the time of irradiation and reconstituted for 8 weeks thereafter. Both males and females were used. Individual experiments were age and sex matched. Mice were bred in specific-pathogen free conditions.

Wild animals

The study did not involve wild animals

Field-collected samples

The study did not involve field-collected samples

Ethics oversight

All experiments described in our study were approved by the "CECCAPP", our local ethics comitte, and were then approved by the French Ministry of Research.

Note that full information on the approval of the study protocol must also be provided in the manuscript.

ChIP-seq

Data deposition

☒ Confirm that both raw and final processed data have been deposited in a public database such as GEO.

☒ Confirm that you have deposited or provided access to graph files (e.g. BED files) for the called peaks.

Data access links
May remain private before publication.

https://www.ncbi.nlm.nih.gov/geo/query/acc.cgi?acc=GSE168242

Files in database submission

GSE168242 Tbet-Eomes Mar 04, 2024 approved None ---
GSE168240 Tbet vs Eomes [RNA-Seq] Mar 04, 2024 approved TXT GSM5134507 NK-Cre CD27- rep1 Mar 04, 2024 approved None GSM5134508 NK-Cre CD11b- rep1 Mar 04, 2024 approved None GSM5134509 Eomes Ho CD27- rep1 Mar 04, 2024 approved None GSM5134510 Eomes Ho CD11b- rep1 Mar 04, 2024 approved None GSM5134511 Tbet WT CD27- rep1 Mar 04, 2024 approved None GSM5134512 Tbet WT CD11b- rep1 Mar 04, 2024 approved None GSM5134513 Tbet Ho CD27- rep1 Mar 04, 2024 approved None GSM5134514 Tbet Ho CD11b- rep1 Mar 04, 2024 approved None GSM5134515 NK-Cre CD27- rep2 Mar 04, 2024 approved None GSM5134516 NK-Cre CD11b- rep2 Mar 04, 2024 approved None GSM5134517 Eomes Ho CD27- rep2 Mar 04, 2024 approved None GSM5134518 Eomes Ho CD11b- rep2 Mar 04, 2024 approved None GSM5134519 Tbet WT CD27- rep2 Mar 04, 2024 approved None GSM5134520 Tbet WT CD11b- rep2 Mar 04, 2024 approved None GSM5134521 Tbet Ho CD27- rep2 Mar 04, 2024 approved None GSM5134522 Tbet Ho CD11b- rep2 Mar 04, 2024 approved None GSM5134523 NK-Cre CD27- rep3 Mar 04, 2024 approved None GSM5134524 NK-Cre CD11b- rep3 Mar 04, 2024 approved None GSM5134525 Eomes Ho CD27- rep3 Mar 04, 2024 approved None GSM5134526 Eomes Ho CD11brep3 Mar 04, 2024 approved None GSM5134527 Tbet WT CD27- rep3 Mar 04, 2024 approved None GSM5134528 Tbet WT CD11b- rep3 Mar 04, 2024 approved None GSM5134529 Tbet Ho CD27- rep3 Mar 04, 2024 approved None GSM5134530 Tbet Ho CD11b- rep3 Mar 04, 2024 approved None ---
GSE168241 T-bet-Eomes [ChIP-Seq] Mar 04, 2024 approved None GSM5134531 WT_1 Mar 04, 2024 approved WIG GSM5134532 WT_2 Mar 04, 2024 approved WIG GSM5134533 Tbet_1 Mar 04, 2024 approved WIG GSM5134534 Tbet_2 Mar 04, 2024 approved WIG GSM5134535 Eomes_1 Mar 04, 2024 approved WIG GSM5134536 Eomes_2 Mar 04, 2024 approved WIG ---

Genome browser session
(e.g. UCSC)

https://www.ncbi.nlm.nih.gov/geo/query/acc.cgi?acc=GSE168242

Methodology

Replicates

two for the ChIP-seq

Sequencing depth

`Sequencing type: paired-end 2x 100bp
Belw: Aligned corresponds to the number of reads aligned only once in the reference genome. Unique corresponds to the number of unique sequences aligned once in the genome. (1) corresponds to mapping results for read 1. (2) corresponds to mapping results for read 2.
Samples
Tbet_1
Tbet_2
Eomes_1
Eomes_2
WT_7
WT_8
Aligned (1)
31554406
35684709
36861749
28630975
26227506
18217573
Unique (1)
20996798
25847152
29584514
23129434
23103920
16433529
Aligned (2)
31089947
35102265
36157801
28104102
25379249
17499027
Unique (2)
20735419
25472620
29041086
22732180
22394227
15803254

Antibodies	anti-HA-Tag (C29F4), Cell Signaling T, Cat# 3724
Peak calling parameters	The Encode Pipeline v1.6.1 was used to map reads and detect Tbet and Eomes peaks. Briefly, bowtie v2.3.4.3 was used to align reads to the reference mouse genome mm10/GRCh38. spp v1.15.5 was used to detect around 300,000 peaks and the IDR method was used to select reproducible peaks with an IDR threshold <= 0.05. The « IDR optimal » set of peaks was selected for downstream analysis. Controls are the mock samples.
Data quality	To ensure reproducibility of the IPs, we compared the number of reads in detected peaks in the replicates. Bedtools [6] makewindows v2.26.0 was used to compute all non overlapping 10Kb long bins along the mouse genome. Bedtools intersect was used to count the number of reads falling into each bin for all IP samples. Read counts per bin are presented in a scatterplot and the Spearman correlation coefficient is computed.<br>Moreover, we compared our data with published data (see details in the methods)
Software	Peaks were annotated relative to genomic fetures using Homer v4.11.1. Pie charts were done using R and the package ggplot2 v3.3.0. Venn diagrams comparing genomic positions (peaks) were created using custom R script using the Bioconductor package DiffBind v2.12.0. Venn diagrams comparing Gene names (here Ensembl Gene IDs) were created using custom R scripts using the R package VennDiagram v1.6.20. Heatmaps were generated using Deeptools v3.5 [9] using the tool bamCoverage to generate bigwigs files with a step of 10 nt. Then, the tool computeMatrix was used to generate a count matrix at the positions of the union of Tbet and Eomes peaks and finally the tools plotHeatmap and plotProfile were used to generate heatmaps and mean profile plots.

Flow Cytometry

Plots

Confirm that:

- [x] The axis labels state the marker and fluorochrome used (e.g. CD4-FITC).
- [x] The axis scales are clearly visible. Include numbers along axes only for bottom left plot of group (a 'group' is an analysis of identical markers).
- [x] All plots are contour plots with outliers or pseudocolor plots.
- [x] A numerical value for number of cells or percentage (with statistics) is provided.

Methodology

Sample preparation	Single-cell suspensions were obtained and stained for surface antigen expression using indicated antibodies for 30 minutes at 4°C. Cell viability was measured using Annexin-V (BD Biosciences)/live-dead fixable (eBiosciences) staining. Intracellular stainings for TFs were performed using Foxp3 kit (eBioscience). Lyse/Fix and PermIII buffers (BD Biosciences) were used for intracellular staining of phosphorylated proteins as described in the manufacturers' data sheet.
Instrument	Flow cytometry was carried out on a FACS LSR II, or a FACS Fortessa (Becton-Dickinson).
Software	Data analysis was performed with FlowJo v.10 (FlowJo LLC)
Cell population abundance	Purity of sorted cell populations was over 98% as checked by flow cytometry.
Gating strategy	All samples were pre-gated using the following strategy: 1) Lymphocytes based on FSC-A/SSC-A; 2)Singlets based on SSC-A/SSC-W. Clear boundaries between negative and positive samples were used to determine gating strategies. Representative plots are shown for all quantified data.

- [x] Tick this box to confirm that a figure exemplifying the gating strategy is provided in the Supplementary Information.
